# Interaction between Digital Economy and Environmental Pollution: New Evidence from a Spatial Perspective

**DOI:** 10.3390/ijerph19095074

**Published:** 2022-04-21

**Authors:** Sa Xu, Cunyi Yang, Zhehao Huang, Pierre Failler

**Affiliations:** 1 School of Economics and Management, Hunan Institute of Technology, Hengyang 421002, China; xusa@hnit.edu.cn; 2 School of Economics and Statistics, Guangzhou University, Guangzhou 510006, China; 2112064077@e.gzhu.edu.cn; 3 Guangzhou Institute of International Finance, Guangzhou University, Guangzhou 510405, China; 4 Centre for Blue Governance, University of Portsmouth, Portsmouth PO1 3DE, UK; pierre.failler@port.ac.uk

**Keywords:** digital economy, green economy, environmental pollution, simultaneous spatial equation, GS3SLS

## Abstract

The digital economy and the green economy are two major issues for economic recovery in the post epidemic era. From spatial interaction spillover, we analyze and measure the relationships between the digital economy and environmental pollution in 287 prefecture-level cities in China from 2008 to 2018 using simultaneous spatial equations and the generalized 3-stage least square (GS3SLS) method. The results show that: (1) there is a reverse and complex spatio-temporal evolution of the digital economy and environmental pollution in Chinese cities. (2) There is a spatial interaction spillover effect between the digital economy and environmental pollution. Local digital economy and environmental pollution inhibit each other. The digital economy and environmental pollution have a significant spatial spillover. The digital economy of surrounding regions has a suppressive effect on local environmental pollution. The environmental pollution of surrounding cities has a crowding-out effect on the local digital economy. (3) Digital economy suppresses environmental pollution through the green development effect and innovative development effect; environmental pollution suppresses the digital economy through the talent crowding out effect and the policy tightening effect. The conclusion of this paper provides evidence for the coupling and coordinated development between the digital and green economy, which is of great significance for promoting the transformation of economic development modes and realizing green and high-quality development.

## 1. Introduction

### 1.1. Motivation

In today’s society, digitization and low-carbon economy are becoming more popular. Digital technology has entered the deepening stage of cross-border integration from the set of knowledge popularization. The digital economy, based on this, has produced substantial economic benefits. In the past, it was generally believed that there was a conflict between economic development and environmental pollution [1,2], but the emergence of the digital economy seemed to break away from this dilemma.

As the largest developing country and carbon emitter, China should pay special attention to reducing pollution and carbon while paying attention to economic development [3,4,5]. In particular, the central government pointed out that “achieving carbon peak by 2030 and striving to achieve carbon neutralization by 2060” put forward higher requirements for pollution reduction in the new era and new stage. It is challenging to achieve green development only by pollution terminal treatment. Effective economic means must form a long-term mechanism for treating environmental pollution. By mixing cutting-edge digital technology such as cloud computing, big data, and artificial intelligence with whole enterprises, the digital economy has challenged existing industries and become a new route of industrial development.

The Chinese central government’s “suggestions on the 14th five-year plan and long-term goals for 2035” put forward a proposal to: “deepen cooperation in public health, digital economy, green development, science, technology, and education, and promote people and cultural exchanges” [6]. The digital economy will progressively become a new driving force in future economic growth, promoting a green economy that can balance the link between the digital economy and environmental resource conservation. China should attach great importance on the changing harmony between the green and digital economy, urging them to join forces and build a new economic growth pattern. As a result, investigating the coupling and coordinated growth link between the green economy and the digital economy is critical for promoting China’s economic development model transition and achieving high-quality green development [7,8,9,10].

**Figure 1:**
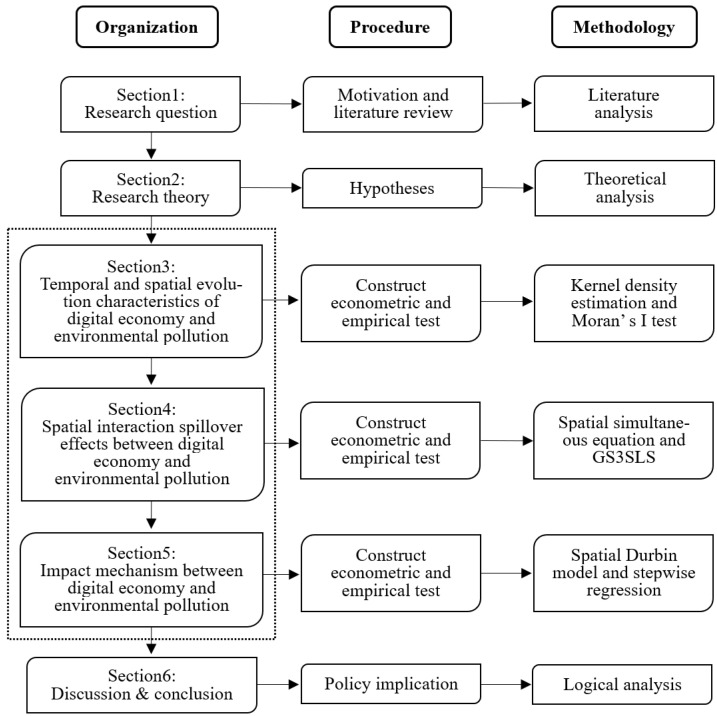
The logical framework.

Based on the above context, this research aims to discuss the link between the growth of the digital economy and environmental pollution by assessing their temporal and geographical evolution features and interaction spillover effects. The textual logic of this research is shown in Figure 1.

The study objective of this article is presented in Section 1, which is based on an examination of the current macroeconomic situation and prior relevant research: how do the digital economy and environmental pollution impact each other from a geographical viewpoint? In Section 2, we develop the research hypothesis of this article and provide the empirical techniques and variable measurements of this study via theoretical analysis. Section 3 examines the features of China’s urban digital economy and environmental pollution in terms of their chronological and geographical development. Section 4 investigates and evaluates the robustness of the spatial interaction spillover effect of China’s urban digital economy and environmental pollution. The interaction mechanism between the digital economy and ecological contamination is examined in Section 5 of this study. The study is summarized in Section 6.

### 1.2. Literature Review and Contribution

There is no consensus on the interaction between digital economy and sustainable development. Extensive studies have shown that the digital economy and green economy are developing in lockstep [11,12,13], with the following categories:

Digitization and sustainable development are the main trends of economic and social development [14,15,16]. Castro et al. reviewed previous studies [17]; they believed that combining digitalization with sustainable development would provide a tremendous opportunity to create a greener economy and society, as well as pave the way for the attainment of sustainable development goals. One of the most potential possibilities for long-term growth is digitization [18]. People are increasingly expecting digital to offer value to achieving sustainable development objectives by providing new data sources, improved analytical skills, and a collaborative digital ecosystem [19,20]. Franca et al. proposed that small cities use blockchain to enhance solid waste management [21].

Emerging digital technologies play a positive role in green development. Innovative digital techniques (such as deep learning and data-driven engineering) may be used to achieve a greater level of automation and early optimization in the design process, which is becoming more vital for the long-term sustainability of resource-based circular economies [22,23]. Rajala et al. explored how smart products may help foster the industrial ecosystem’s long-term growth [24]. The research contributes to our understanding of the circular economy’s closed-loop system, which today relies more than ever on the digital platform. Dumont et al. believe that digital technology is low-cost and easy to obtain, which provides power for sustainable ecological agriculture [25].

The digital sharing economy is considered to be green and environmentally protective. The sharing economy platform improves the reuse rate of existing commodities and alleviates unnecessary resource consumption [26]. Digital development promotes the popularity of shared travel, which encourages consumers to use shared transportation instead of buying private cars [26,27,28]. Compared with taxis, digital ride services have better energy efficiency and reduced greenhouse gas emissions. Uber’s mileage capacity usage rate (61%) was greater than that of taxis (49.1%), according to Cramer and Krueger by using the trip data of taxis and Uber in five cities in the United States [29].

Besides, the inverse relationship between economic growth and pollution has been explored in the abundant literature on Environmental Kuznets Curve (EKC), including the spatial effect and impact of the digital economy [30,31]. When economic development reaches a specific point, the “inflection point”, environmental degradation tends to go from high to low, the pollution intensity steadily decreases, and ecological protection is improved [32]. This theory has been confirmed by a large number of studies and exists in countries worldwide [33], especially in China [34,35].

However, many studies have proposed in a tense relationship between digitization and sustainable development. According to Martin et al. [36], there are five conflicts between smart cities and urban sustainable development goals, and environmental protection is neglected. The significance of a shared digital economy on energy consumption and greenhouse gas emissions, according to Jin et al., is so far unknown [37]. While replacing taxis, digital ride service also hurts other green travel modes, such as cycling, public transport, walking, and carpooling. By analyzing six European cities’ digitization and carbon footprint, Akande et al. found a contradiction between smart cities and sustainable development [38]. They believe that “a city can be smart but not sustainable and vice versa”. Kuntsman and Rattle propose that digital equipment has caused great damage to the environment during production, maintenance, and scrapping [39].

Compared with previous studies, this paper provides new evidence for the synergy between the digital economy and green development from advanced spatial interaction. The following is a list of the critical work and minor contributions: Build China’s urban digital economy and environmental pollution index from several dimensions and study their temporal and geographic development features of them, using samples from more than 200 prefecture-level and above cities in China, as well as historical data from 2008 to 2018. The following study examines the spatial interaction spillover impact of China’s urban digital economy and environmental pollution using simultaneous spatial equations and the generalized three-stage least square technique (GS3SLS) and ultimately investigate the interaction mechanism between them. The empirical results show that: (1) between China’s urban digital economy growth and environmental pollution, there is a degree of reverse development trend and complicated geographical distribution. (2) The urban digital economy and environmental pollution in China have a spatial interaction spillover effect. Local digital economy and pollution can hurt each other. The digital economy and environmental pollutants have a substantial regional spillover impact. The digital economy of surrounding cities inhibits local environmental pollution, and the environmental pollution of surrounding cities has no significant crowding-out effect on the local digital economy. (3) The digital economy suppresses environmental pollution through the green development effect and innovative development effect; environmental pollution suppresses the digital economy through the talent crowding out effect and the policy tightening effect.

## 2. Research Design

### 2.1. Hypothesis

China’s digital economy is widely assumed to be on a long-term and steady upward trajectory. China’s digital economy has increased in recent years. As per the white paper on China’s digital economy growth (2020), the magnitude of China’s digital economy added value has spread from 2.6 trillion yuan in 2005 to 35.8 trillion yuan in 2019. From 14.2 per cent in 2005 to 36.2 per cent in 2019, its share of GDP has grown. The fast expansion of the local economy has tremendously aided the prosperity of the area’s digital economy. The economic growth of Chinese cities, on the other hand, is highly imbalanced, resulting in an unequal temporal and geographical distribution of the digital economy’s development. Similarly, as the Chinese government places a greater emphasis on environmental preservation, pollution should steadily decrease. However, due to regional economic imbalances, there are variances in the elimination pace of various non-green enterprises (such as heavy pollution and mining) in different areas, resulting in complicated environmental pollution evolution features across cities; evidence from Europe can provide a reference for this [40]. The north and south of the EU region can be understood as China’s coastal and inland areas. Both parts have established emission trading markets. It can be explained that the source of growth is technological innovation, which brings new digital technology and leads to the production of new industrial products. Similarly, emission reduction quotas will reduce pollution emissions in China, reduce pollution in coastal areas, and increase pollution in inland regions. As a result, we propose the following hypothesis:

**Hypothesis** **1** **(H1).** *In Chinese cities, the digital economy and pollution have reversed and complicated temporal and geographical dynamic development features.*

The digital economy and environmental damage have a complicated connection. The first is the effect of digitalization on pollution levels. Since the neoclassical growth model was proposed, academic circles have unanimously agreed that technological advancement favors efficiency. Boosting the expansion of the digital economy would encourage the intensive change in the industrial production model by enabling technical innovation to reduce pollution. Besides, the digital economy can transform Internet traffic’s value into economic and ecological value and provide technical reserves and product application incentives for green consumption. Using digital technology to develop green consumer items and create a green consumption platform may increase public engagement and feeling of ownership in green consumption and enhance the communication efficiency of the green consumption idea. However, it is also possible that increased production due to improved efficiency will increase emissions. For example, in R&D-based growth model, such as the Romer model, when material capital is a pollution source, technological progress in the form of variety increase can induce rising pollution emissions by increasing material capital. Even if technological progress is achieved by putting pollution-reducing products into the production of final products, pollution will not decrease. The same is true when the source of growth is human capital. Therefore, technological progress can reduce pollution when the final products produced by improving efficiency are put into the production of pollution-reducing products or linked with ecological innovation that helps reduce pollution [41,42]. The second point to consider is the influence of pollution on the digital economy. The reform and opening since 1978 have brought opportunities for China’s economic development. However, the extensive economy dominated by heavy industry has also caused a massive burden on the ecological environment. China has steadily become one of the most polluted nations due to its past policy of focusing on growth rather than conservation. Environmental and ecological concerns have become more significant as China’s industrialization process has accelerated. Among them, the frequent occurrence of air pollution is the most apparent embodiment, which directly affects the health of most residents and continuously causes various social and economic losses. As an essential part of today’s social and economic development, the digital economy has also been damaged. However, the digital economy and environmental pollution do not only interact within the city as their specific spatial distribution characteristics; rather, there is a complex spatial interaction spillover effect. As a result, we propose the second hypothesis:

**Hypothesis** **2** **(H2).** *There is a spatial interaction spillover effect between the digital economy and environmental pollution in Chinese cities.*

**Figure 2:**
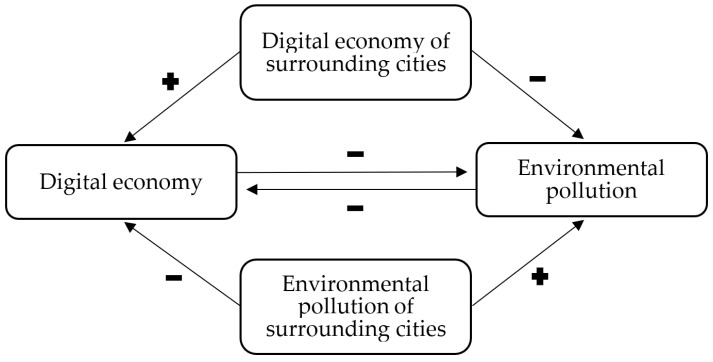
The link between the digital economy and pollution in the environment.

Figure 2 shows the structure of H2.

Ecological efficiency and innovation are the two main transmission channels for the digital economy’s impact on green development. The digital economy is a new economic structure that has emerged due to technical advancements in electronic equipment, communication networks, and data processing. The digital economy and its information communication channels will significantly improve the efficiency of knowledge dissemination, which will undoubtedly enhance the knowledge stock of the whole economy in the macro dimension, and then drive technological innovation. In reality, for example, big data and Internet of Things (IoT) platforms can effectively improve the efficiency of enterprise information collection and integration and then help enterprises in developing new products. This innovation driver promotes economic efficiency while also having a substantial beneficial influence on environmental efficiency. From the reverse inhibition of environmental pollution in the digital economy, the extrusion of talents and a harsh policy environment may be the primary transmission mechanism. In the long-term process of industrialization, the environmental damage in some developed cities in China is severe, and high pollution problems such as haze are challenging to solve with speed. The willingness to “escape” from the town is gradually strengthened for professionals who pay attention to health. “Air pollution is becoming a greater concern for our members and their families.” Tang Yadong, Secretary-General of the European Chamber of Commerce in China, once said, “although there are many reasons for members to leave here, we almost always hear that air pollution is one of the reasons.” A reduction in emission allowances would trigger technological innovation and affect the location of polluting firms [42]. This would impact the health of the population in the background. The relocation of firms can also be interpreted as the migration of talent if the location of polluting companies is accompanied by the migration of researchers and technicians working with the developed technologies [43,44,45]. Furthermore, China’s central government has consistently imposed environmental protection duties on local governments in recent years, and appropriate laws and regulations have been published one after the other [46,47,48]. Some stringent laws have even irreversibly harmed local businesses. In this situation, rules may potentially stifle the growth of the digital economy. Therefore, we put forward the third hypothesis:

**Hypothesis** **3** **(H3).** *Ecological and innovation variables have a mediating role in the digital economy’s ability to reduce pollution. Talent flow and regulatory variables mediate the reverse inhibition of environmental degradation in the digital economy.*

### 2.2. Model

According to this paper’s theoretical analysis and research assumptions, there is a link between the digital economy and the degree of environmental contamination. As a result, a fixed effect panel regression model based on simultaneous equations is developed as follows:

(1) $$\mathrm{d\_ec}o_{it}=\alpha_{0}+\alpha_{1}ep_{it}+\alpha X_{it}+\pi_{i}+\varepsilon_{it}$$

(2) $$ep_{it}=\beta_{0}+\beta_{1}\mathrm{d\_ec}o_{it}+\beta Z_{it}+\mu_{i}+\sigma_{it}$$

where i is the city individual and t is the time; d_eco and ep, respectively, represent the digital economy and the pollution in the sample cities; $X_{it}$ and $Z_{it}$ are the control variables that affect the digital economy and the pollution; $\pi_{i}$ and $\mu_{i}$ represent the fixed effect; $\varepsilon_{it}$ and $\sigma_{it}$ represent the error term.

Using the usual fixed effects panel model to estimate the content of this article results in some mistakes. To begin with, the geographical link between the degree of digital economy growth and the amount of pollution is overlooked. That is, the impact of nearby cities on smaller cities is overlooked. Previous scientists have often used classic spatial econometric models to handle this challenge. The Spatial Lag Model (SLM), the Spatial Error Model (SEM), and the Spatial Dobbin Model (SDM) are examples of models that look at the one-way effects of significant variables. The spatial interplay between them was not well analyzed [49]. As a result, this research develops a simultaneous spatial equation to characterize the spatial interaction of the urban digital economy with pollution. Second, the possibility of a link between endogenous factors and random disturbance terms is overlooked. As a result, the spatial interaction spillover effect between the level of digital economy growth and the degree of environmental pollution was investigated using Theil and Zellner’s generalized spatial 3-stage least square approach (GS3SLS) [50]. The simultaneous spatial equations are set as:

(3) $$\mathrm{d\_ec}o_{it}=\alpha_{0}+\alpha_{1}\overset{n}{\underset{j\neq i}{\sum }}W\mathrm{d\_ec}o_{it}+\alpha_{2}\overset{n}{\underset{j\neq i}{\sum }}Wep_{it}+\alpha_{3}ep_{it}+\alpha X_{it}+\varepsilon_{it}$$

(4) $$ep_{it}=\beta_{0}+\beta_{1}\overset{n}{\underset{j\neq i}{\sum }}Wep_{it}+\beta_{2}\overset{n}{\underset{j\neq i}{\sum }}W\mathrm{d\_ec}o_{it}+\beta_{3}\mathrm{d\_ec}o_{it}+\beta Z_{it}+\sigma_{it}$$

W is the spatial weight matrix in Equations (3) and (4). $X_{it}$ and $Z_{it}$ are the control variables that affect the digital economy and the pollution, including economic development level, urbanization, industrial structure, openness, marketization, population density, transportation, and posts. Moreover, there is no need to set the fixed effect, which has been confirmed by a large number of studies in the past [49,51,52]. The spatial weight matrix of geographical distance ($W_{1}$) and economic-geographical distance ($W_{2}$) are built, respectively, due to the complexity of spatial spillovers. $W_{1}$ uses the latitude and longitude coordinates of each city center to determine the straight-line distance between them. Take the reciprocal as the weight and set the bandwidth to 0–30 using dimensionless processing. If the distance between the two cities’ centers exceeds 30, the weight value is set to 0, indicating that the two cities are not nearby. $W_{2}$ examines the economic distance between cities based on geographical distance. We calculate individual elements in the matrix using the following methods:

(5) $$EC\mathrm{O\_G}E\mathrm{O\_D}istance_{i,j}=GE\mathrm{O\_D}istance_{i,j}\times EC\mathrm{O\_D}istance_{i,j}$$

$EC\mathrm{O\_G}E\mathrm{O\_D}istance_{i,j}$ reflects the economic-geographical distance between cities i and j, whereas $GE\mathrm{O\_D}istance_{i,j}$ represents the geographical distance between cities i and j in Equation (5). The absolute magnitude of the per capita GDP difference between cities i and j is represented by $EC\mathrm{O\_D}istance_{i,j}$. The normalized weight is then taken as the reciprocal of $EC\mathrm{O\_G}E\mathrm{O\_D}istance_{i,j}$.

$\alpha_{1}$ represents the overflow strength and direction of the digital economy development level from the nearby city. $\beta_{1}$ represents the overflow strength and direction of the environmental pollution from the nearby city. The variables $\alpha_{2}$ and $\beta_{2}$ are utilized to validate the geographical connection between digital economics and pollution. The former indicates the degree and direction of neighboring urban environment pollution’s influence on the local digital economy development level; the latter indicates the magnitude and direction of the impact of adjacent cities’ digital economy development levels on local environmental degradation. The endogenous link between the digital economy and the degree of pollution is described by $\alpha_{3}$ and $\beta_{3}$.

The GS3SLS is utilized in this study for a global estimate based on the simultaneous spatial equations listed above. The degree of environmental degradation and the amount of digital economy growth are endogenous variables in the spatial simultaneous equation model. The use of OLS estimation will result in parameter estimation inconsistency. The GS3SLS is used to estimate the overall space, the estimation method and considering the spatial correlation and the endogenous variable potential equation of random perturbation terms possible correlation between problems, improve the effectiveness of the estimation results.

In addition, the above theoretical mechanism analysis shows that the green development effect and innovative development effect are the two main mechanisms of the digitization economy affecting environmental pollution. In comparison, the two main mechanisms of environmental pollution affecting the digital economy are the talent crowding effect and the policy tightening effect. This paper adopts the mediating effect model based on spatial analysis to verify these four mechanisms to conduct empirical research [53]. The particular model is established as follows, using the impact mechanism of the digital economy on environmental degradation as an example:

(6) $$\mathrm{d\_ec}o_{it}=\alpha_{0}+\alpha_{1}\overset{n}{\underset{j\neq i}{\sum }}W\mathrm{d\_ec}o_{it}+\alpha_{2}\overset{n}{\underset{j\neq i}{\sum }}Wep_{it}+\alpha_{3}ep_{it}+\alpha X_{it}+\varepsilon_{it}$$

(7) $$M_{it}=\beta_{0}+\beta_{1}ep_{it}+\beta \;X_{it}+\sigma_{it}$$

(8) $$\mathrm{d\_ec}o_{it}=\gamma_{0}+\gamma_{1}\overset{n}{\underset{j\neq i}{\sum }}W\;\mathrm{d\_ec}o_{it}+\gamma_{2}\overset{n}{\underset{j\neq i}{\sum }}W\;ep_{it}+\gamma_{3}ep_{it}+\gamma_{4}M_{it}+\gamma \;X_{it}+\mu_{it}$$

Equation (6) is the same as Equation (3) above, and the meanings of various variables are consistent with the above. M is the mediating variable, including urban ecological efficiency and regional innovation and entrepreneurship index. When analyzing the influence mechanism of environmental pollution on the digital economy, M includes the number of scientific researcher and the environmental regulation index. Section 2.3 shows the specific economic meaning and sources.

### 2.3. Variables

According to the maximum availability of data, the sample for this article includes 287 prefecture-level and above cities in China, with a temporal dimension ranging from 2008 to 2018.

Firstly, the core variables of this paper are the digital economy (d-eco) and environmental pollution (ep). The entropy weight approach is used in this study to calculate the digital economy’s development level based on three factors [54]. The first indicator is digital finance index, obtained by the Python web crawler method based on the practices of Su et al. [55], and Yao et al. [56]. The other two indicators are digital industry (consisting of employees in the digital industry and total telecommunication services), and digital infrastructure that consists of internet penetration and mobile phone penetration. The weight of each index measured by the entropy weight method is shown in Table 1. Its essence is to use the entropy of variables to estimate the amount of information contained in variables. The digital industry and digital finance are the predominant drivers of the urban digital economy. There are obvious differences in the development of the digital industry and digital finance among regions. However, with the gradual popularization of digital equipment, there is no significant difference in the level of digital infrastructure among areas, then its proportion is also low. In this paper, the degree of environmental pollution includes three common types of pollution: wastewater, waste gas, and smoke. Per capita wastewater pollutants (t/person), industrial sulfur dioxide pollutants (t/person), and industrial smoke emissions (t/person) are the units of measurement. The data are from China’s city statistical yearbook. Then, Z-score is used for standardization, addition, and normalization.

Secondly, the mediating variables of this paper are urban ecological efficiency (e-eff), regional innovation and entrepreneurship index (inn), the number of researchers (rd), and environmental regulation index (er). The DEA model, which is based on the super-efficient SBM-GML model, is used to assess urban ecological efficiency [57]. Fixed assets, employment, urban area, and energy usage are the input indicators. GDP is the anticipated production, and the unexpected output is wastewater, waste gas, and smoke [58,59]. The regional innovation and entrepreneurship index comes from the Center for Enterprise Research of Peking University. The number of scientific researchers comes from China’s city statistical yearbook, represented by the number of scientific researchers every 10 thousand people and normalized [60]. The fourth mediating variable is the environmental regulation index, which is analyzed by Chen et al. [61]. Specifically, this paper uses the annual government work reports of cities at all levels in China to analyze the proportion of statements containing keywords of environmental regulation in the full text. The keywords are 45 Chinese words such as “environmental protection, environmental protection, green development, new energy, haze…”. After using text analysis to measure the proportion of environmental regulation-related sentences, we multiplied by 100 and took the first-order difference to obtain the environmental regulation index of prefecture-level cities in China.

Lastly, eight core variables-related control variables were chosen [62,63,64,65]. We took into account the city’s per capita GDP and logarithm it (pgdp); Regional urbanization level (urb) is the fraction of the city’s population living in the municipal area; The share of secondary and tertiary industries in the city’s GDP (ind); Openness (ope), the city’s real foreign investment share of GDP; The level of marketization (mar), the fraction of private and individual employment in the whole population; Take the logarithm of the population density (den) of the whole city; Total city passenger volume divided by total population, expressed as a logarithm (tra); Postal development level (pos), revenue from postal business divided by the entire population.

Table 2 lists the variables’ sources and descriptive statistics.

## 3. Spatio-Temporal Evolution of Digital Economy and Environmental Pollution

### 3.1. Temporal Evolution

**Figure 3:**
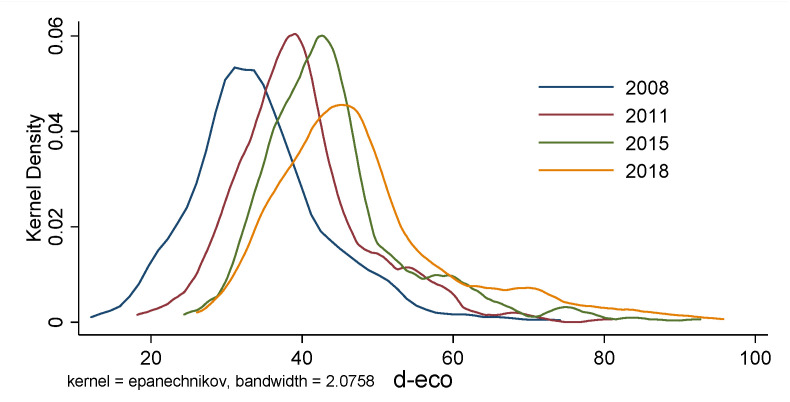
Temporal evolution of the digital economy.

**Figure 4:**
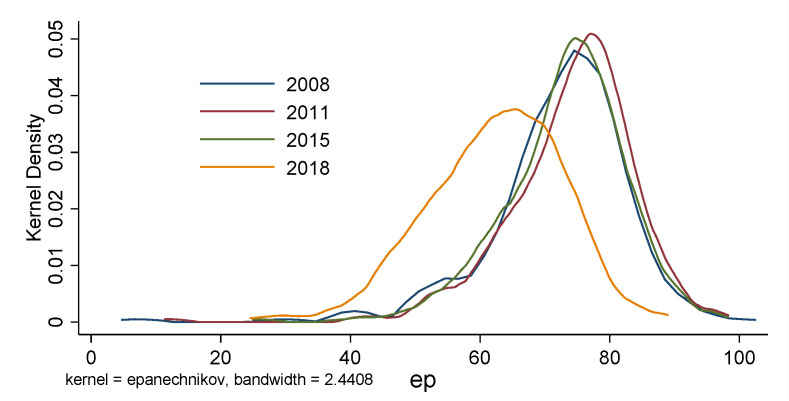
Temporal evolution of environmental pollution.

Using the nonparametric kernel density estimation calculation Equation and Stata 16.0 software, the global kernel density curves of China’s urban digital economy level and environmental pollution in 2008, 2011, 2015, and 2018 are drawn (see Figure 3 and Figure 4). In probability theory, kernel density estimation is used to estimate the unknown density function. It belonged to one of the nonparametric test methods and was proposed by Rosenblatt (1955) and Emanuel Parzen (1962), also known as the Parzen window [66].

The global scale kernel density analysis can depict the temporal evolution of China’s urban digital economy and pollution level. (1) From 2008 to 2011, 2011 to 2015, and 2015 to 2018, the center of gravity of the digital economy shifted to the right, indicating that the degree of the urban digital economy in China grew over the study period. In terms of pollution, there is no notable shift in emphasis from 2008 to 2015, and a solid left movement from 2015 to 2018, demonstrating that China’s cities have achieved tremendous accomplishments in environmental protection in recent years. (2) The prominent peak of the curve displays a tendency of first rising and then declining, demonstrating that the digital economic gap between cities is first widening and then closing. In terms of pollution, there was no notable change from 2008 to 2015 and a clear decreasing trend from 2015 to 2018, showing that the pollution gap across Chinese cities has narrowed in recent years. (3) The number of curve peaks indicates no multipolar differentiation pattern in the urban digital economy and pollution. (4) The digital economy’s tailing on the right is more significant than its tailing on the left. The number of cities in high-value regions has grown, whereas the number of cities in low-value areas has declined. The left tail of the pollution degree curve is longer and thicker than the right tail, showing that urban pollution in low-value locations has grown. However, the share of low-value cities has risen. The digital economy’s growth level, polarization degree, and pollution in Chinese cities have various evolution features.

### 3.2. Spatial Evolution

#### 3.2.1. Global Spatial Evolution

**Figure 5:**
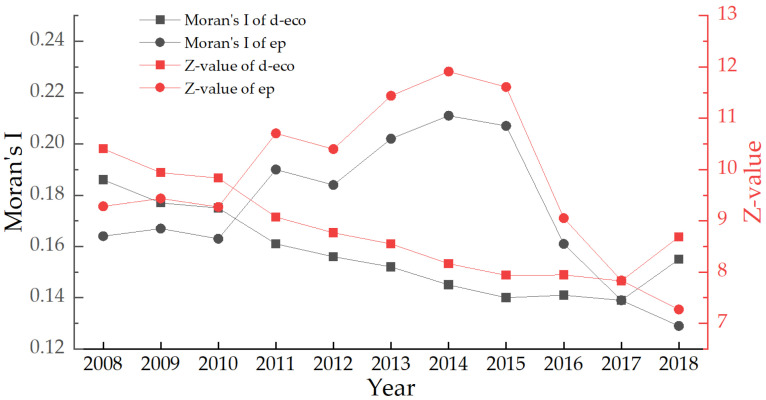
Global spatial evolution of digital economy and environmental pollution in China.

Using the global Moran index and GeoDa software, this section estimates the global Moran’s I and Z-values of China’s urban digital economy and environmental pollution from 2008 to 2018. (see Figure 5). Moran’s I is a measure of spatial autocorrelation developed by Patrick Alfred Pierce Moran [67,68,69,70]. In short, it determines whether there is a correlation between spatial entities in a specific range.

In terms of the digital economy, the global Moran’s I is positive, ranging from 0.139 to 0.186, and the Z-value is between 7.827 and 10.403, all of which pass the 1% significance test. The geographical distribution of China’s urban digital economy development level showed a substantial positive global spatial autocorrelation, and the surrounding cities impacted the local cities’ digital economy development. Overall, China’s urban digital economy growth level shows a weakening geographical relationship. In terms of pollution, the global Moran’s I range from 0.129 to 0.211, and the Z-value ranges from 7.272–11.911. China’s environmental pollution had a solid positive global spatial autocorrelation over the study period, with local pollution impacted by pollution in nearby cities. Overall, the global geographic connection of pollution in Chinese cities shows solid and weak associations. The geographical association rose between 2008 and 2014, then dropped between 2014 and 2018.

#### 3.2.2. Regional Spatial Evolution

**Figure 6:**
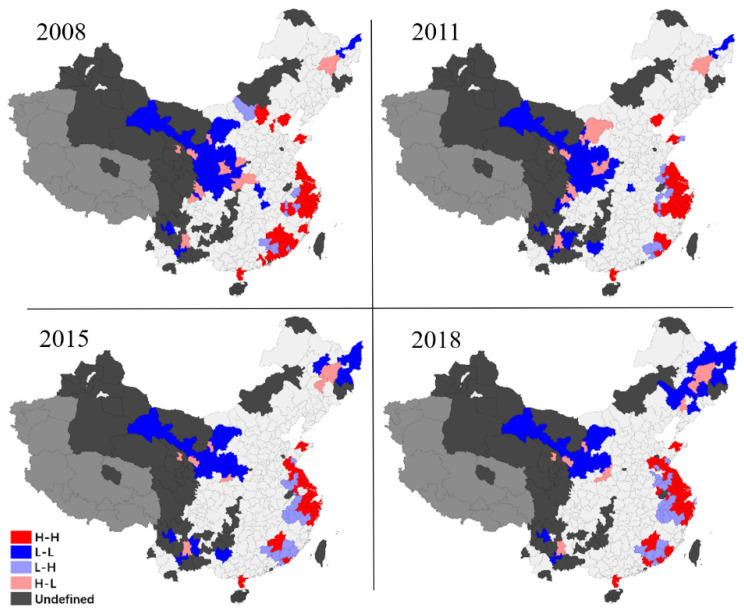
Regional Evolution of spatial patterns of the digital economy.

**Figure 7:**
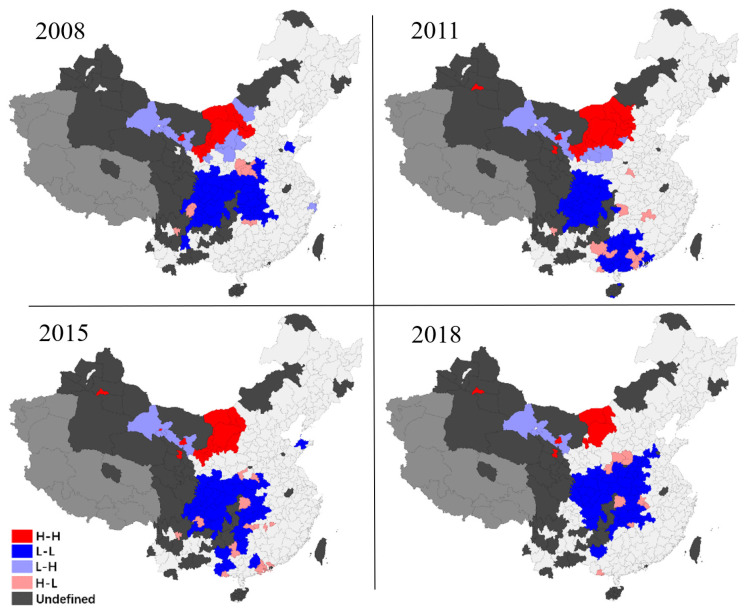
Regional Evolution of spatial patterns of environmental pollution.

The global Moran index can show that there is global spatial autocorrelation between digital economy growth and pollution in Chinese cities. However, if we want to study local geographical aspects, we need to identify them using local spatial autocorrelation (Figure 6 and Figure 7). This section uses the Local Indicators of Spatial Association (LISA) method to characterize local areas’ digital economy and pollution [71]. The figures on this page are for simple distribution presentation only and cannot be used as maps. In the digital economy, for example, the local spatial pattern may be split into four categories: In the first category, agglomeration type “high-high,” high-level spatial equilibrium associated agglomeration state “high center and high periphery” suggests high-level digital economy growth in local cities and adjacent cities; The second is “low-low” agglomeration. This kind shows a low-level spatial equilibrium linked agglomeration state of “low center and low periphery” with a low-level digital economy development level in local cities; The third variety is “low-high.” This class reflects a low degree of digital economy growth in local cities. However, the neighboring cities’ digital economy development levels are high, indicating a “low center and high periphery” agglomeration condition; Another agglomeration type is “high-low.” This kind depicts the geographical imbalance of “high center and low periphery” in the digital economy.

Figure 6 shows a complex local spatial pattern in the urban digital economy in China. In terms of the distribution of spatial patterns, it is evident that there are extensive “L-L” and “H-L” development patterns in Western and Northeast China, which shows that in areas with relatively backward economic growth in China, the digital economy is also hindered. Key cities plunder the digital economy resources of surrounding average towns. In the developed areas along the eastern coast, there is a large “H-H” and “L-H” development pattern, which shows sufficient resource connectivity among cities with better development in areas with the developed digital economy in China. In contrast, cities with the slow growth of the digital economy in developed regions are vulnerable to being siphoned by surrounding cities. In terms of the evolution of spatial pattern, the distribution of “L-L” and “H-L” in the western region is reduced, which means that the digital economy in Western China is positive. However, the “L-L” distribution and diffusion in Northeast China and the growth pattern of the digital economy continues to deteriorate. The digital economy growth pattern in the developed areas along the eastern coast remains unchanged.

Figure 7 shows that there is also an unbalanced local spatial pattern of urban environmental pollution in China. In terms of geographical pattern distribution, western and central China have wide “L-L” and “H-L” growth patterns, indicating that environmental pollution in these areas is generally low. A few industrial cities absorb the pollution emission pressure of surrounding cities. There is a small area of “H-H” and “L-H” development patterns in the northwest, indicating an aggregation effect of environmental pollution in this area. In terms of the evolution of spatial pattern, the distribution of “L-L” and “H-L” in the midwestern regions diffused during the investigation period, which means that the environmental pollution across the central and western parts of China has been alleviated, and the green development interacts among cities. The distribution of “H-H” in Northwest China has shrunk, and the environmental pollution has been alleviated to a certain extent.

## 4. Spatial Interaction Spillover Effects of the Digital Economy and the Environmental Pollution

### 4.1. Parameter Estimation Results

The first phase simply evaluates the link between the digital economy and the environmental pollution value in Table 3 using the benchmark model Equations (1) and (2).

Table 3 reveals that the digital economy and pollution have a significant mutual inhibitory impact. Columns (1) and (4) demonstrate the scenarios when the individual is not controlled, and the control variable is not considered. Columns (2) and (5) depict the situation in which the individual is not held, but the control variable is considered. Columns (3) and (6) show the case in which the individual is controlled while the control variable is also considered. As seen in Columns (1)–(3), the digital economy is hampered by environmental degradation. The digital economy performs a reverse inhibitory function in environmental contamination, as seen in Columns (4)–(6).

In the second step, we analyze the spatial interaction effect and spillover effect between the digital economy and environmental pollution using simultaneous spatial equations and GS3SLS in Table 4.

The digital economy and environmental contamination have a solid geographical interaction spillover impact, as shown in Table 4. The following are the particular outcomes: Column (1) shows that the surrounding cities’ digital economy development level has a beneficial influence on the local digital economy’s development level; environmental pollution in the surrounding town has no discernible inhibitory impact on the degree of growth of the regional digital economy; local environmental pollution hurts the local digital economy’s development level. Column (2) shows that the surrounding cities’ digital economy development level has an inhibitory effect on local environmental pollution; the surrounding cities’ environmental pollution promotes local environmental pollution; and the local digital economy’s development level has an inhibitory effect on local environmental pollution. Furthermore, the coefficients may be compared if the significance is checked and the economic importance is the same. The negative impact of local environmental pollution on the development level of the local digital economy is smaller than the inhibiting effect of environmental pollution in neighboring cities on the development level of the local digital economy. The green impact of local digital economy growth is consistent with the inhibitory effect of neighboring cities’ digital economy development levels on local environmental degradation. The findings are essentially the same economic-geographical distance, as seen in columns (3) and (4).

### 4.2. Results Analysis

#### 4.2.1. General Interaction Effect between Digital Economy and Environmental Pollution

This research discovers a reciprocal inhibitory relationship through parameter estimation between the degree of the digital economy and environmental contamination. In terms of pure geographical distance, the level of the digital economy fell by 0.173 (*p* = 0.057) for each unit of increased environmental degradation. The environmental pollution was reduced by 0.133 (*p* = 0.072) for each unit of the digital economy. Overall, there is an interactive effect between the digital economy level and environmental pollution, with the marginal effect of environmental pollution on the digital economy level being more apparent. In the reciprocal promotion connection between the two, the marginal impact of environmental pollution on the digital economy level is comparatively dominating. The digital economy consists of a set of economic activities that help improve efficiency and optimize the economic structure, as well as various infrastructure and services that can support the digitization of economic activities; the green economy aspires for the growth of the economy, society, and environment in a coordinated manner. It’s a well-balanced economy that encompasses a variety of classic industrial economies. The digital and green economies work closely together and interact as the two most crucial new economic forms in the future. Environmental degradation also has hurt the digital economy’s growth.

#### 4.2.2. Spatial Spillover Effect between Digital Economy and Environmental Pollution

This article discovers considerable regional spillovers in the digital economy and environmental damage through parameter estimation. From a pure geographical distance standpoint, the local digital economy grows by 0.129 (*p* = 0.035) for every unit of growth in the digital economy level of neighboring cities. The digital economies of neighboring cities may have a favorable impact on the development of the local digital economy via industrial external expansion radiation and the shared market effect. For each unit of environmental pollution in surrounding cities, the environmental pollution in local cities increases significantly by 0.999 (*p* = 0.000). The aggregation of polluting industries often accompanies the increase in environmental pollution in surrounding cities. Transportation costs induce enterprises to form upstream and downstream industrial clusters in nearby areas, and the pollution coverage is gradually expanded to improve the environmental pollution in local cities.

#### 4.2.3. Spatial Interaction Effect between Digital Economy and Environmental Pollution

According to parameter estimates, the surrounding digital economy has an inhibiting impact on local environmental pollution. In contrast, the increase in environmental pollution in surrounding cities has an extrusion effect on the local digital economy level. In terms of pure geographical distance, each unit of rising in the digital economy level of neighboring cities reduces local environmental pollution by 0.133 (*p* = 0.001). The spread of the digital economy to neighboring cities has brought green development resources and environmental protection technology spillover, in turn engineering urban growth and building. Cities with lagging green economy growth should welcome, with an open mind, the spillover support of adjacent cities’ digital economy development to the local green economy while modernizing their sectors. The local digital economy level may decline by 0.101 (*p* = 0.656) for each unit of environmental pollution in adjacent cities. Environmental pollution has prominent diffusion characteristics. For example, air pollution in industrial cities will quickly spread to surrounding cities. Taking Hebei Province of China as an example, some cities with severe industrial pollution have brought serious haze to the region, including many underdeveloped agricultural areas. The increase in environmental pollution in surrounding cities negatively impacts local digital economy investment and digital economy talents, resulting in an obvious crowding out effect. Underdeveloped areas of the digital economy should take advantage of surrounding cities’ digital economy diffusion mechanism to actively carry out mild green transformation and alleviate the environmental pressure caused by industrialization.

### 4.3. Robustness Test

The empirical findings in Section 4.2 are based on the spatial geographic weight matrix and the economic-geographic weight matrix, which have a bandwidth of 0–30. The matrix context may be unique, making it challenging to reach persuasive and general results. As a result, in this part, we use empirical data to assess the universality of the bandwidth and kind of geographical geographic weight matrix.

First, we reduce the bandwidth of W in Equations (3) and (4) from 0–30 to 0–20 and expand to 0–40. At this time, a single city sample will have fewer or more “neighbors.” The bandwidth robustness test parameter estimate results are shown in Table 5.

Table 5 shows that the regression findings are stable and that changing the distance band setting has no effect on the parameter estimation results. First, the level of the digital economy and environmental pollution have a mutually inhibitory impact; second, the digital economy and environmental pollution have significant spatial spillovers; third, the level of the surrounding digital economy has an inhibitory effect on local ecological pollution, and the increase in environmental pollution in surrounding cities has also squeezed out the status of the local digital economy.

Secondly, there are usually two forms of the spatial weight matrix; one is a quantitative matrix in which the weight is equal to the reciprocal of the distance between samples. The alternative option is to use a qualitative weight matrix to split the distance between samples into “neighbor relationship” and “non neighbor relationship” categories. We change the type of W in Equations (3) and (4) from the first to the second. At this time, the spatial relationship between sample cities is only 0 (non-adjacent) and 1 (adjacent). Table 6 shows the matrix type robustness test parameter estimation results, and the distance bandwidth thresholds are set to 0–20, 0–30, and 0–40.

The regression findings are still robust, as shown in Table 6, and the way of modifying the spatial weight matrix has no effect on the parameter estimation outcomes.

## 5. Additional Analysis

The findings of the above empirical investigation demonstrate a complicated geographical connection between the digital economy and pollution, which inhibits each other. This article will perform an empirical analysis utilizing the mediating effect model to understand better the relationship between the digital economy and environmental degradation. According to the theoretical mechanism analysis, the green development impact and the inventive development effect are the two critical mechanisms for the digital economy to influence the growth of environmental degradation. The two primary methods for ecological degradation to harm the digital economy are the talent crowding out effect and the policy tightening effect. The mechanisms above will be empirically tested in this section.

The stepwise test regression coefficient approach based on spatial analysis is used to assess the mediating function of urban ecological efficiency in the inhibition of digital economy on environmental pollution, as shown in Equations (6) and (8). Table 7 displays the regression findings.

By observing Table 7, it can be found that the digital economy has a significant inhibitory effect on environmental pollution through the green development effect. From the direction and significance of the coefficient in Column (1), it can be seen that the digital economy has a large negative influence on environmental contamination, which is consistent with the benchmark regression result; the influence of the digital economy on urban ecological efficiency is verified in Column (2), and the influence coefficient is 0.003, which means that digital economy encourages the growth of ecological efficiency; the impact coefficient of the local digital economy on environmental pollution in Column (3) is −0.012, which is lower than the impact coefficient of the local digital economy on environmental pollution in Column (1). The impact coefficient of intermediary variable urban ecological efficiency on environmental pollution is −1.035, which is significant at the 1% confidence level, which means that improving urban ecological efficiency will reduce environmental pollution. To summarize, the inhibitory effect of the digital economy on environmental pollution is partly realized by promoting ecological efficiency. Similarly, when the regression findings of Columns (4)–(6) are combined, the inhibitory impact of the digital economy on environmental pollution is partially achieved through enhancing urban ecological efficiency from the standpoint of economic-geographical distance.

By observing Table 8, it can be found that the digital economy has a significant inhibitory effect on environmental pollution through the effect of innovation development. Columns (1)–(3) show that the inhibitory effect of the digital economy on environmental pollution is partly realized by promoting regional innovation and entrepreneurship index. Similarly, the regression results of Columns (4)–(6) show that from the standpoint of economic-geographical distance, the inhibitory effect of the digital economy on environmental pollution is partly realized by promoting regional innovation and entrepreneurship index.

By observing Table 9, it can be found that the digital economy is significantly hampered by pollution through the talent crowding out effect. Columns (1)–(3) show that the inhibitory effect of environmental pollution on the digital economy is partly realized by reducing the number of scientific researchers. Similarly, the regression findings in Columns (4)–(6) reveal that the inhibitory impact of environmental pollution on the digital economy is partially achieved by limiting the number of scientific researchers from the aspect of economic-geographical distance.

By observing Table 10, it can be found that the digital economy is significantly hampered by pollution through the tightening effect of the policy. Columns (1)–(3) show that the inhibitory effect of environmental pollution on the digital economy is partly realized by strengthening environmental regulations. Similarly, the regression results of Columns (4)–(6) show that from the standpoint of economic-geographical distance, the inhibitory effect of environmental pollution on the digital economy is partly realized by strengthening environmental regulations.

## 6. Discussions

There is a complex interaction mechanism between the urban digital economy and the degree of environmental pollution. The special spatial distribution characteristics make the spatial interaction spillover effect between them need to be deeply explored. This paper uses multi-dimensional indicators to calculate the digital economy and environmental pollution degree of more than 200 cities in China from 2008 to 2018. It analyzes their temporal and spatial evolution characteristics, spatial interaction spillover effect, and interaction mechanism. Through empirical analysis, we obtain the following significant findings:

Firstly, reverse and complex spatio-temporal evolution of the digital economy and environmental pollution in Chinese cities. (1) The entire growth of China’s urban digital economy is increasing with time. The disparity in development levels widens and then narrows. The number and development level of head cities have grown. Between 2015 and 2018, total environmental contaminants decreased significantly. The pollution difference between cities has narrowed recently. The number of clean zones increased, but so did their pollution. (2) China’s urban digital economy and pollution distribution show significant geographical autocorrelation in global spatial evolution. The spatial correlation of the digital economy shows a fluctuating evolution from strong correlation to weak correlation, and the spatial correlation of environmental pollution shows the evolution characteristics of strong alternating correlation and weak correlation. (3) In terms of regional spatial evolution, the digital development in Western and Northeast China is generally weak, and the eastern coastal areas have the “alliance between giants” Development pattern. Some key cities siphon digital economic resources in the surrounding backward areas. There is a certain degree of pollution accumulation in Northwest China, while environmental pollution in the central and western regions is relatively light. A few industrial cities absorb the pollution emission pressure of surrounding cities. The analysis of temporal and spatial evolution characteristics shows that there is a high imbalance between China’s digital economy and environmental pollution, which is consistent with the previous research results in other regions. The unbalanced development will inevitably lead to the continuous flow of factors between regions, which leads to the subsequent conclusion.

Secondly, there is a complex spatial interaction spillover effect between the digital economy and environmental pollution. (1) The digital economy and pollution are mutually inhibited. The digital economy contributes to ecologically friendly development, while pollution impacts the local digital economy. (2) The digital economy and pollution have enormous geographical spillover effects. The digital economy will spread to the neighboring regions, raising the urban circle’s total digital level. The industrial agglomeration of polluting enterprises leads to the correlation of regional emissions. (3) The level of the surrounding digital economy can restrain the local environmental pollution, and the increase in environmental pollution in surrounding cities can also squeeze out the local digital economy. The radiation of the digital economy to surrounding cities has brought an overflow of green development resources and environmental protection technology. In contrast, the diffusion of pollution emissions with air and other media has caused the outflow of emerging digital industries in surrounding cities. The development of digital economy has crossed the “inflection point” of EKC and formed a “joint force” with environmental protection, promoting each other and developing in a virtuous circle. However, the results of spatial interaction spillover effect show that there is diffusion and competition between digital economy and environmental pollution outside or between cities, and the previous research focusing on the research within the sample is not enough.

Finally, we found the specific interaction mechanism between the digital economy and environmental pollution. (1) The digital economy reduces pollution by encouraging green and creative growth. The vibrant digital economy has led to emerging digital technologies and platforms and significantly promoted ecological efficiency. Technological innovation and efficiency improvement are of crucial significance to green development. (2) Environmental contamination hinders the digital industry by talent outflow and restricting policies. Serious pollution problems such as haze in industrialized cities lead to losing high-tech talents. The relevant environmental laws and regulations issued to make up for the ecological deterioration also hurt economic development. These are the inevitable consequences of “pollution first and then treatment.”

Admittedly, this study also has several deficiencies. (1) Even though this paper employs a spatial econometric method to investigate the relationship between the digital economy and environmental pollution, its conclusion is based on historical data and econometric reasoning, lacks in-depth economic theoretical analysis, and can only reflect the evolution of data characteristics and their relationships during the study period. This disadvantage may have an impact on the study’s long-term effectiveness. In the future, we should develop a universal economic theoretical model to assess the usefulness of the results of this work and make appropriate projections. (2) It should be noted that the covariates in this paper are selected according to the availability of data and the mainstream methods of previous research, which is less targeted to the digital economy and environmental pollution topics in this paper. More relevant variables should be actively constructed for analysis if further research can be carried out. (3) Although this paper uses the cutting-edge spatial econometric model, there are still two common shortcomings. One is that the primary estimation method of the spatial econometric model is the Maximum Likelihood Estimate (MLE), but the large sample theory of MLE needs to be improved. The other is that we must subjectively set a non-random spatial weight matrix instead of estimating the matrix according to the data. The spatial weight matrix may not be entirely reflected the complex relationship between different regions. Although many robustness tests have been carried out in this paper to minimize this error, it is unavoidable. (4) Due to the data availability limit, this study uses index variables for analyses. It would largely constrain policy implications potentially linked to the results. (5) The last deficiency is the sample problem of this paper. According to Figure 6 and Figure 7, readers can find that some cities in central and Western China do not consider it due to the lack of data. In the empirical test, it also impacts the effectiveness of the test of spatial interaction spillover effect.

## 7. Conclusions

The conclusion of this paper serves as a point of reference for many nations’ and regions’ digital economy and green development initiatives. According to the analysis of temporal and spatial evolution characteristics, it is necessary to balance the digital economy and the share of environmental degradation across areas and equitably divide digital economy development resources so that backward regions may also benefit from high-quality economic growth enabled by the digital economy. Environmental pollution enterprises should also be appropriately distributed to avoid areas with excessive pollution concentration from breaking through the upper limit of ecological self-regulation capacity and causing irreversible ecological damage. The significant spatial spillover effect shows that to grow cities in underdeveloped regions, it needs to learn from adjacent cities’ experiences with digital economy usage and management and cut down or green-update backward sectors; the negative spatial interaction warns us that, while consolidating their development, advanced areas of the digital economy and green development should use their resources to help neighboring backward cities carry out green innovation and pollution control, improve the inhibition ability of the digital economy to environmental pollution, weaken the reverse containment of environmental pollution to economic development, and play an exemplary and leading role. The digital economy is a new engine to accelerate economic development and a new idea to promote social high-quality green development. Finally, according to the apparent influence path of this paper, the government should improve the digital economy infrastructure, pay attention to the development of scientific and technological talents, consolidate the foundations of digital industrialization, use the digital economy as a key starting point for upgrading industrial structure, assist businesses in improving their level of digital technology innovation, and accelerate the transformation of digital scientific and technological achievements in various fields.

## Figures and Tables

**Table 1:** Digital economy indicator system.

Primary Indicator	Secondary Indicator	Weight
Digital finance (0.3169)	Digital finance index	0.3169
Digital industry (0.5818)	Employees in digital industry	0.3508
	Total Telecommunication Services	0.2310
Digital infrastructure (0.1013)	Internet penetration	0.0367
	Mobile phone penetration	0.0646

**Table 2:** Data sources and descriptive statistics.

Variable	Abbr.	Unit	Items	Summary	Source
Digital economy	d-eco	-	Mean Std	41.925 10.724	Entropy weight method
Environmental pollution	ep	-	Mean Std	6.027 6.854	Statistical yearbook
Ecological efficiency	e-eff	-	Mean Std	1.528 0.516	Data Envelopment Analysis
Innovation and entrepreneurship index	inn	-	Mean Std	52.588 28.465	Center for Enterprise Research
Number of scientific researchers	rd	-	Mean Std	42.963 13.051	Statistical yearbook
Environmental regulation index	er	-	Mean Std	6.933 2.742	Textual analysis
Economic Development Level	pgdp	ln(RMB/person)	Mean Std	10.498 0.644	Statistical yearbook
Urbanization	urb	ratio	Mean Std	0.357 0.238	Statistical yearbook
Industrial Structure	ind	ratio	Mean Std	0.872 0.081	Statistical yearbook
Openness	ope	ratio	Mean Std	0.027 0.096	Statistical yearbook
Marketization	mar	ratio	Mean Std	0.128 0.136	Statistical yearbook
Population density	den	ln(person/km^2^)	Mean Std	5.725 0.917	Statistical yearbook
Transportation	tra	ln(RMB/person)	Mean Std	2.771 0.784	Statistical yearbook
Posts	pos	ln(RMB/person)	Mean Std	4.235 0.796	Statistical yearbook

**Table 3:** Benchmark regression results.

Items	d-eco			ep		
	(1)	(2)	(3)	(4)	(5)	(6)
d-eco	-	-	-	−0.065 *** (−5.70)	−0.305 *** (−22.04)	−0.055 *** (−2.65)
ep	−0.159 *** (−5.70)	−0.442 *** (−22.04)	−0.045 *** (−2.65)	-	-	-
pgdp	-	6.669 *** (21.18)	10.079 *** (44.05)	-	3.161 *** (11.53)	0.048 (0.15)
urb	-	−1.386 ** (−2.19)	3.551 *** (4.35)	-	5.225 *** (10.10)	0.764 (0.84)
ind	-	12.160 *** (5.29)	−9.973 *** (−3.64)	-	24.079 *** (12.89)	5.059 * (1.66)
ope	-	−3.623 *** (−3.03)	−0.648 (−1.19)	-	−1.995 ** (−2.01)	−0.396 (−0.66)
mar	-	17.961 *** (15.89)	13.038 *** (15.28)	-	4.960 *** (5.10)	−1.199 (−1.22)
den	-	2.637 *** (18.21)	1.160 ** (2.46)	-	−1.348 *** (−10.85)	−4.514 *** (−8.75)
tra	-	0.125 (0.77)	−0.610 *** (−5.54)	-	1.184 *** (8.83)	1.629 *** (13.71)
pos	-	2.309 *** (12.42)	1.056 *** (7.94)	-	−0.355 ** (−2.25)	−0.691 *** (−4.65)
N	3124	3124	3124	3124	3124	3124
FE	No	No	Yes	No	No	Yes
R2	0.0103	0.6414	0.7296	0.0103	0.3936	0.1705
F	32.52 ***	618.95 ***	848.91 ***	32.52 ***	224.62 ***	64.66 ***

Notes: ***, **, and * stand for significant levels of 1%, 5%, and 10%, respectively. Bracket values are T-values.

**Table 4:** Global estimation results of GS3SLS.

Items	Geographical Distance		Economic–Geographical Distance	
	d-eco (1)	ep (2)	d-eco (3)	ep (4)
W× d-eco	0.129 ** (2.10)	−0.133 *** (−3.30)	0.053 * (1.75)	−0.037 (−1.34)
W× ep	−0.101 (−0.45)	0.999 *** (11.80)	−0.063 (−1.10)	0.010 (0.18)
d-eco	-	−0.133 * (−1.80)	-	−0.926 *** (−14.48)
ep	−0.173 * (−1.86)	-	−0.745 *** (−13.58)	-
pgdp	5.361 *** (9.75)	3.187 *** (6.83)	6.837 *** (17.73)	6.664 *** (13.59)
urb	−2.438 ** (−2.43)	4.723 *** (8.35)	0.797 (1.12)	2.890 *** (4.67)
ind	9.358 *** (2.77)	17.787 *** (9.21)	19.526 *** (7.62)	26.545 *** (12.71)
ope	−2.553 ** (−2.06)	−1.724 * (−1.74)	−3.792 *** (−3.19)	−3.931 *** (−3.60)
mar	17.714 *** (15.05)	2.634 * (1.67)	17.614 *** (15.48)	15.713 *** (9.83)
den	3.226 *** (9.91)	−1.522 *** (−5.39)	1.823 *** (9.67)	0.864 *** (3.33)
tra	−0.048 (−0.24)	0.566 *** (4.15)	0.615 *** (3.48)	0.869 ** (5.71)
pos	2.584 *** (11.94)	−0.249 (−1.02)	1.842 *** (9.38)	1.450 *** (5.86)
N	3124	3124	3124	3124
R2	0.9503	0.6822	0.9796	0.3651
F	5002.69 ***	535.88 ***	13297.45 ***	343.36 ***

Notes: ***, **, and * stand for significant levels of 1%, 5%, and 10%, respectively. Bracket values are T-values.

**Table 5:** Results of bandwidth adjustment.

Items	Bandwidth: 0 to 20		Bandwidth: 0 to 40	
	d-eco	ep	d-eco	ep
	(1)	(2)	(3)	(4)
W× d-eco	0.244 *** (4.55)	−0.263 *** (−6.57)	0.166 ** (2.51)	−0.173 *** (−4.15)
W× ep	−0.075 (−0.65)	0.599 *** (8.13)	−0.559 ** (−2.34)	1.045 *** (11.82)
d-eco	-	−0.102 *** (−2.80)	-	−0.050 *** (2.66)
ep	−0.048 * (−1.86)	-	−0.237 * (1.66)	-
pgdp	4.829 *** (3.54)	2.927 *** (6.02)	4.315 *** (7.82)	2.326 *** (4.91)
urb	−3.116 *** (5.21)	4.416 *** (7.61)	−4.658 *** (−4.63)	5.428 *** (9.43)
ind	5.750 * (1.70)	16.176 *** (8.23)	2.588 (0.77)	16.716 *** (8.54)
ope	−2.778 ** (−3.17)	−1.595 (−1.57)	−1.902 (−1.49)	−1.237 (−1.23)
mar	17.301 *** (14.39)	0.629 (0.39)	17.738 *** (14.53)	−0.601 (−0.38)
den	3.479 *** (10.79)	−1.913 *** (−6.56)	4.075 *** (12.74)	−2.190 *** (−7.60)
tra	−0.135 (0.13)	0.753 *** (5.46)	−0.315 (−1.53)	0.564 *** (4.04)
pos	2.606 *** (12.35)	−0.746 *** (−2.94)	2.863 *** (12.97)	−0.723 *** (−2.90)
N	3124	3124	3124	3124
R2	0.9780	0.6547	0.9248	0.6196
F	12,492.64 ***	533.57 ***	3905.33 ***	491.66 ***

Notes: ***, **, and * stand for significant levels of 1%, 5%, and 10%, respectively. Bracket values are T-values.

**Table 6:** Results of the matrix type adjustment.

Items	Bandwidth: 0 to 20 (Contiguity)		Bandwidth: 0 to 30 (Contiguity)		Bandwidth: 0 to 40 (Contiguity)	
	d-eco	ep	d-eco	ep	d-eco	ep
	(1)	(2)	(3)	(4)	(5)	(6)
W× d-eco	0.448 *** (10.10)	−0.362 *** (−5.77)	0.217 *** (4.34)	−0.086 ** (−2.11)	0.656 *** (6.62)	−0.041 (−1.18)
W× ep	0.081 (0.91)	0.224 *** (2.73)	−0.734 *** (5.13)	0.896 *** (17.51)	−0.271 *** (−3.96)	0.918 *** (18.16)
d-eco	-	−0.033 (0.23)	-	−0.434 *** (−4.77)	-	−0.551 *** (−8.01)
ep	−0.234 ** (−2.44)	-	−0.626 *** (−4.24)	-	−1.802 *** (−7.17)	-
pgdp	3.763 *** (7.93)	2.889 *** (4.13)	6.242 *** (11.64)	4.674 *** (9.26)	9.370 *** (11.13)	5.182 *** (12.19)
urb	−4.705 *** (−5.74)	5.211 *** (7.43)	0.116 (0.11)	4.217 *** (7.10)	6.849 *** (4.04)	3.814 *** (6.80)
ind	4.484 (1.54)	15.655 *** (6.79)	19.229 *** (5.42)	21.663 *** (10.79)	41.188 *** (7.19)	22.830 *** (11.70)
ope	−1.670 (−1.31)	−1.987 * (−1.92)	−2.983 ** (−2.46)	−2.405 ** (−2.50)	−4.977 *** (−2.91)	−2.753 *** (−2.86)
mar	17.798 *** (14.88)	−0.450 (−0.17)	18.286 *** (16.20)	8.281 *** (4.46)	18.649 *** (11.63)	10.281 *** (6.73)
den	4.246 *** (16.36)	−2.423 *** (−4.59)	2.166 *** (5.67)	−0.832 ** (−2.36)	−0.738 (−1.15)	−0.423 (−1.54)
tra	−0.155 (−0.84)	0.803 *** (5.77)	0.236 (1.28)	0.460 *** (3.72)	0.866 *** (3.49)	0.480 *** (3.81)
pos	2.749 *** (12.92)	−0.859 ** (−2.12)	2.293 *** (11.02)	0.593 ** (2.13)	1.620 *** (5.37)	0.889 *** (3.79)
N	3124	3124	3124	3124	3124	3124
R2	0.9878	0.5909	0.9884	0.8858	0.9889	0.8725
F	24,709.48 ***	435.11 ***	22,685.97 ***	2066.63 ***	27,343.34 ***	1967.06 ***

Notes: ***, **, and * stand for significant levels of 1%, 5%, and 10%, respectively. Bracket values are T-values.

**Table 7:** Mechanism test of green development effect.

Items	Geographical Distance			Economic–Geographical Distance		
	ep	M	ep	ep	M	ep
	(1)	(2)	(3)	(4)	(5)	(6)
d-eco	−0.014 ** (−2.49)	0.003 *** (2.95)	−0.012 ** (−2.33)	−0.035 * (−1.74)	0.003 *** (2.95)	−0.034 * (−1.72)
W× d-eco	−0.203 *** (−6.05)	-	−0.162 *** (−4.70)	−0.230 *** (−7.82)	-	−0.175 *** (−5.93)
W× ep	0.845 *** (24.73)	-	0.800 *** (20.43)	0.304 *** (11.53)	-	0.263 *** (9.81)
M	-	-	−1.035 *** (−6.53)	-	-	−1.619 *** (−9.98)
Controls	Yes	Yes	Yes	Yes	Yes	Yes
N	3124	3124	3124	3124	3124	3124
R2	0.1776	0.2425	0.2231	0.2050	0.2425	0.2351

Notes: ***, **, and * stand for significant levels of 1%, 5%, and 10%, respectively. Bracket values are T-values.

**Table 8:** Mechanism test of innovative development effect.

Items	Geographical Distance			Economic–Geographical Distance		
	ep	M	ep	ep	M	ep
	(1)	(2)	(3)	(4)	(5)	(6)
d-eco	−0.014 ** (−2.49)	1.564 *** (36.15)	−0.013 * (1.86)	−0.035 * (−1.74)	1.564 *** (36.15)	−0.033 * (−1.65)
W× d-eco	−0.203 *** (−6.05)	-	−0.206 *** (−5.97)	−0.230 *** (−7.82)	-	−0.208 *** (−7.37)
W× ep	0.845 *** (24.73)	-	0.844 *** (24.67)	0.304 *** (11.53)	-	0.291 *** (10.21)
M	-	-	−0.005 * (1.69)	-	-	−0.008 * (−1.75)
Controls	Yes	Yes	Yes	Yes	Yes	Yes
N	3124	3124	3124	3124	3124	3124
R2	0.1776	0.6569	0.1779	0.2050	0.6569	0.2050

Notes: ***, **, and * stand for significant levels of 1%, 5%, and 10%, respectively. Bracket values are T-values.

**Table 9:** Mechanism test of talent crowding out effect.

Items	Geographical Distance			Economic–Geographical Distance		
	d-eco	M	d-eco	d-eco	M	d-eco
	(1)	(2)	(3)	(4)	(5)	(6)
ep	−0.111 *** (−6.61)	−0.617 *** (−19.44)	−0.108 *** (−6.33)	−0.022 ** (−2.40)	−0.617 *** (−19.44)	−0.018 ** (−2.12)
W× ep	−0.106 ** (−2.22)	-	−0.017 (−0.35)	−0.110 *** (−3.51)	-	−0.103 *** (−3.12)
W× d-eco	0.850 *** (52.57)	-	0.754 *** (41.46)	0.358 *** (16.13)	-	0.329 *** (15.08)
M	-	-	0.196 *** (11.86)	-	-	0.206 *** (13.77)
Controls	Yes	Yes	Yes	Yes	Yes	Yes
N	3124	3124	3124	3124	3124	3124
R2	0.7277	0.3947	0.7298	0.7533	0.3947	0.7530

Notes: *** and ** stand for significant levels of 1% and 5%, respectively. Bracket values are T-values.

**Table 10:** Mechanism test of policy tightening effect.

Items	Geographical Distance			Economic–Geographical Distance		
	d-eco	M	d-eco	d-eco	M	d-eco
	(1)	(2)	(3)	(4)	(5)	(6)
ep	−0.111 *** (−6.61)	0.031 *** (3.82)	−0.108 *** (−6.38)	−0.022 ** (−2.40)	0.031 *** (3.82)	−0.016 ** (1.99)
W× ep	−0.106 ** (−2.22)	-	−0.101 ** (−2.11)	−0.110 *** (−3.51)	-	−0.108 *** (−3.45)
W× d-eco	0.850 *** (52.57)	-	0.852 *** (52.23)	0.358 *** (16.13)	-	0.348 *** (15.63)
M	-	-	−0.051 ** (2.04)	-	-	−0.024 *** (−3.90)
Controls	Yes	Yes	Yes	Yes	Yes	Yes
N	3124	3124	3124	3124	3124	3124
R2	0.7277	0.1016	0.7280	0.7533	0.1016	0.7540

Notes: *** and ** stand for significant levels of 1% and 5%, respectively. Bracket values are T-values.

## Data Availability

The data presented in this study are available on request from the author.

## References

[1] Lu Z.-N., Chen H., Hao Y., Wang J., Song X., Mok T.M. (2017). The dynamic relationship between environmental pollution, economic development and public health: Evidence from China. J. Clean. Prod..

[2] Liang W., Yang M. (2019). Urbanization, economic growth and environmental pollution: Evidence from China. Sustain. Comput. Inform. Syst..

[3] Liao G., Drakeford B.M. (2019). An analysis of financial support, technological progress and energy efficiency:evidence from China. Green Financ..

[4] Zheng Y., Chen S., Wang N. (2020). Does financial agglomeration enhance regional green economy development? Evidence from China. Green Financ..

[5] Hao Y., Zheng S., Zhao M., Wu H., Guo Y., Li Y. (2020). Reexamining the relationships among urbanization, industrial structure, and environmental pollution in China—New evidence using the dynamic threshold panel model. Energy Rep..

[6] Guan C., Weng Y., Zhao J., Lin Y., Zhang W., Tu Q. (2021). Examining China’s sustainable development based on genuine progress indicator. Sustain. Prod. Consum..

[7] Li Z., Ao Z., Mo B. (2021). Revisiting the Valuable Roles of Global Financial Assets for International Stock Markets: Quantile Coherence and Causality-in-Quantiles Approaches. Mathematics.

[8] Li Z., Chen L., Dong H. (2021). What are bitcoin market reactions to its-related events?. Int. Rev. Econ. Financ..

[9] Li Z., Dong H., Floros C., Charemis A., Failler P. (2021). Re-examining Bitcoin Volatility: A CAViaR-based Approach. Emerg. Mark. Finance Trade.

[10] Li Z., Huang Z., Failler P. (2022). Dynamic Correlation between Crude Oil Price and Investor Sentiment in China: Heterogeneous and Asymmetric Effect. Energies.

[11] Kostoska O., Kocarev L. (2019). A Novel ICT Framework for Sustainable Development Goals. Sustainability.

[12] Vinuesa R., Azizpour H., Leite I., Balaam M., Dignum V., Domisch S., Felländer A., Langhans S.D., Tegmark M., Nerini F.F. (2020). The role of artificial intelligence in achieving the Sustainable Development Goals. Nat. Commun..

[13] Wu J., Guo S., Huang H., Liu W., Xiang Y. (2018). Information and Communications Technologies for Sustainable Development Goals: State-of-the-Art, Needs and Perspectives. IEEE Commun. Surv. Tutor..

[14] Ahi K., Laidroo L. (2019). Banking market competition in Europe-financial stability or fragility enhancing?. Quant. Financ. Econ..

[15] Qamruzzaman M., Wei J. (2019). Do financial inclusion, stock market development attract foreign capital flows in developing economy: A panel data investigation. Quant. Financ. Econ..

[16] Tripathy N. (2019). Does measure of financial development matter for economic growth in India?. Quant. Financ. Econ..

[17] Del Río Castro G., Fernández M.C.G., Colsa U. (2021). Unleashing the convergence amid digitalization and sustainability towards pursuing the Sustainable Development Goals (SDGs): A holistic review. J. Clean. Prod..

[18] Gouvea R., Kapelianis D., Kassicieh S. (2018). Assessing the nexus of sustainability and information & communications technology. Technol. Forecast. Soc. Change.

[19] Sachs J.D., Schmidt-Traub G., Mazzucato M., Messner D., Nakicenovic N., Rockstroem J. (2019). Six Transformations to achieve the Sustainable Development Goals. Nat. Sustain..

[20] Seele P., Lock I. (2017). The game-changing potential of digitalization for sustainability: Possibilities, perils, and pathways. Sustain. Sci..

[21] França A., Neto J.A., Gonçalves R., Almeida C. (2019). Proposing the use of blockchain to improve the solid waste management in small municipalities. J. Clean. Prod..

[22] Gan V.J.L., Lo I.M.C., Ma J., Tse K.T., Cheng J.C., Chan C.-M. (2020). Simulation optimisation towards energy efficient green buildings: Current status and future trends. J. Clean. Prod..

[23] Hao J.L., Cheng B., Lu W., Xu J., Wang J., Bu W., Guo Z. (2020). Carbon emission reduction in prefabrication construction during materialization stage: A BIM-based life-cycle assessment approach. Sci. Total Environ..

[24] Rajala R., Hakanen E., Mattila J., Seppala T., Westerlund M. (2018). How Do Intelligent Goods Shape Closed-Loop Systems?. Calif. Manag. Rev..

[25] Dumont B., Groot J.C.J., Tichit M. (2018). Review: Make ruminants green again—How can sustainable intensification and agroecology converge for a better future?. Animal.

[26] Frenken K., Schor J. (2017). Putting the sharing economy into perspective. Environ. Innov. Soc. Transit..

[27] Cervero R., Golub A., Nee B. (2007). City CarShare: Longer-term travel demand and car ownership impacts. Transp. Res. Rec..

[28] Firnkorn J., Mueller M. (2015). Free-floating electric carsharing-fleets in smart cities: The dawning of a post-private car era in urban environments?. Environ. Sci. Policy.

[29] Cramer J., Krueger A.B. (2016). Disruptive Change in the Taxi Business: The Case of Uber. Am. Econ. Rev..

[30] Maddison D. (2006). Environmental Kuznets curves: A spatial econometric approach. J. Environ. Econ. Manag..

[31] Zhong K. (2022). Does the digital finance revolution validate the Environmental Kuznets Curve? Empirical findings from China. PLoS ONE.

[32] DE Bruyn S.M. (1997). Explaining the environmental Kuznets curve: Structural change and international agreements in reducing sulphur emissions. Environ. Dev. Econ..

[33] Shuai C., Chen X., Wu Y., Zhang Y., Tan Y. (2019). A three-step strategy for decoupling economic growth from carbon emission: Empirical evidences from 133 countries. Sci. Total Environ..

[34] Song Y., Sun J., Zhang M., Su B. (2020). Using the Tapio-Z decoupling model to evaluate the decoupling status of China’s CO2 emissions at provincial level and its dynamic trend. Struct. Change Econ. Dyn..

[35] Wu Y., Tam V.W.Y., Shuai C., Shen L., Zhang Y., Liao S. (2019). Decoupling China’s economic growth from carbon emissions: Empirical studies from 30 Chinese provinces (2001–2015). Sci. Total Environ..

[36] Martin C.J., Evans J., Karvonen A. (2018). Smart and sustainable? Five tensions in the visions and practices of the smart-sustainable city in Europe and North America. Technol. Forecast. Soc. Change.

[37] Jin S.T., Kong H., Wu R., Sui D.Z. (2018). Ridesourcing, the sharing economy, and the future of cities. Cities.

[38] Akande A., Cabral P., Casteleyn S. (2019). Assessing the Gap between Technology and the Environmental Sustainability of European Cities. Inf. Syst. Front..

[39] Kuntsman A., Rattle I. (2019). Towards a Paradigmatic Shift in Sustainability Studies: A Systematic Review of Peer Reviewed Literature and Future Agenda Setting to Consider Environmental (Un)sustainability of Digital Communication. Environ. Commun.—J. Nat. Cult..

[40] Hamaguchi Y. (2021). Environmental policy effects: An R&D-based economic growth model with endogenous labour supply. J. Econ. Policy Reform.

[41] Chu H., Lai C.C. (2014). Abatement R&D, market imperfections, and environmental policy in an endogenous growth model. J. Econ. Dyn. Control..

[42] Hamaguchi Y. (2021). Polluting firms’ location choices and pollution havens in an R&D-based growth model for an international emissions trading market. J. Int. Trade Econ. Dev..

[43] Nakada M. (2004). Does Environmental Policy Necessarily Discourage Growth?. J. Econ..

[44] Grimaud A., Tournemaine F. (2007). Corrigendum to “Why can an environmental policy tax promote growth through the channel of education?”. Ecol. Econ..

[45] Grimaud A. (1999). Pollution Permits and Sustainable Growth in a Schumpeterian Model. J. Environ. Econ. Manag..

[46] Liu C., Xin L., Li J. (2022). Environmental regulation and manufacturing carbon emissions in China: A new perspective on local government competition. Environ. Sci. Pollut. Res..

[47] Wang C.a., Liu X., Xi Q., Zhang Y. (2022). The Impact of Emissions Trading Program on the Labor Demand of Enterprises: Evidence From China. Front. Environ. Sci..

[48] Xin L., Sun H., Xia X., Wang H., Xiao H., Yan X. (2022). How does renewable energy technology innovation affect manufacturing carbon intensity in China?. Environ. Sci. Pollut. Res..

[49] Gan T., Yang H., Liang W. (2020). How do urban haze pollution and economic development affect each other? Empirical evidence from 287 Chinese cities during 2000–2016. Sustain. Cities Soc..

[50] Zellner A., Theil H. (1962). Three-Stage Least Squares: Simultaneous Estimation of Simultaneous Equations. Econometrica.

[51] Gebremariam G.H., Gebremedhin T.G., Schaeffer P.V. (2010). Analysis of county employment and income growth in Appalachia: A spatial simultaneous-equations approach. Empir. Econ..

[52] Long R., Gan X., Chen H., Wang J., Li Q. (2020). Spatial econometric analysis of foreign direct investment and carbon productivity in China: Two-tier moderating roles of industrialization development. Resour. Conserv. Recycl..

[53] Hayes A. (2009). Beyond Baron and Kenny: Statistical Mediation Analysis in the New Millennium. Commun. Monogr..

[54] Wang S., Yang C., Li Z. (2021). Spatio-Temporal Evolution Characteristics and Spatial Interaction Spillover Effects of New-Urbanization and Green Land Utilization Efficiency. Land.

[55] Su Y., Li Z., Yang C. (2021). Spatial Interaction Spillover Effects between Digital Financial Technology and Urban Ecological Efficiency in China: An Empirical Study Based on Spatial Simultaneous Equations. Int. J. Environ. Res. Public Health.

[56] Yao Y., Hu D., Yang C., Tan Y. (2021). The impact and mechanism of fintech on green total factor productivity. Green Financ..

[57] Yang C., Li T., Albitar K. (2021). Does Energy Efficiency Affect Ambient PM2.5? The Moderating Role of Energy Investment. Front. Environ. Sci..

[58] Yang X., Wang W., Wu H., Wang J., Ran Q., Ren S. (2021). The impact of the new energy demonstration city policy on the green total factor productivity of resource-based cities: Empirical evidence from a quasi-natural experiment in China. J. Environ. Plan. Manag..

[59] Zhao S., Cao Y., Feng C., Guo K., Zhang J. (2022). How do heterogeneous R&D investments affect China’s green productivity: Revisiting the Porter hypothesis. Sci. Total Environ..

[60] Wang M., Li Y., Liao G. (2021). Research on the Impact of Green Technology Innovation on Energy Total Factor Productivity, Based on Provincial Data of China. Front. Environ. Sci..

[61] Chen Z., Kahn M.E., Liu Y., Wang Z. (2018). The consequences of spatially differentiated water pollution regulation in China. J. Environ. Econ. Manag..

[62] Li F., Yang C., Li Z., Failler P. (2021). Does Geopolitics Have an Impact on Energy Trade? Empirical Research on Emerging Countries. Sustainability.

[63] Li T., Li X., Liao G. (2021). Business cycles and energy intensity. Evidence from emerging economies. Borsa Istanb. Rev..

[64] Li Z., Zou F., Mo B. (2021). Does mandatory CSR disclosure affect enterprise total factor productivity?. Econ. Res.—Ekon. Istraz..

[65] Li Z., Zou F., Tan Y., Zhu J. (2021). Does Financial Excess Support Land Urbanization-An Empirical Study of Cities in China. Land.

[66] Botev Z.I., Grotowski J.F., Kroese D.P. (2010). Kernel Density Estimation via Diffusion. Ann. Stat..

[67] Moran P.A.P. (1950). Notes on continuous stochastic phenomena. Biometrika.

[68] Li H., Calder C.A., Cressie N. (2007). Beyond Moran’s I: Testing for spatial dependence based on the spatial autoregressive model. Geogr. Anal..

[69] Yang X., Wang J., Cao J., Ren S., Ran Q., Wu H. (2021). The spatial spillover effect of urban sprawl and fiscal decentralization on air pollution: Evidence from 269 cities in China. Empir. Econ..

[70] Liu P., Zhang L., Tarbert H., Yan Z. (2022). Analysis on Spatio-Temporal Characteristics and Influencing Factors of Industrial Green Innovation Efficiency-From the Perspective of Innovation Value Chain. Sustainability.

[71] Yin L., Wang L., Huang W., Liu S., Yang B., Zheng W. (2021). Spatiotemporal Analysis of Haze in Beijing Based on the Multi-Convolution Model. Atmosphere.

